# Formation of Transient Lamellipodia

**DOI:** 10.1371/journal.pone.0087638

**Published:** 2014-02-05

**Authors:** Juliane Zimmermann, Martin Falcke

**Affiliations:** 1 Max-Delbrück-Center for Molecular Medicine, Mathematical Cell Physiology, Berlin, Germany; 2 Center for Theoretical Biological Physics, Rice University, Houston, Texas, United States of America; Karolinska Institutet, Sweden

## Abstract

Cell motility driven by actin polymerization is pivotal to the development and survival of organisms and individual cells. Motile cells plated on flat substrates form membrane protrusions called lamellipodia. The protrusions repeatedly appear and retract in all directions. If a lamellipodium is stabilized and lasts for some time, it can take over the lead and determine the direction of cell motion. Protrusions traveling along the cell perimeter have also been observed. Their initiation is in some situations the effect of the dynamics of the pathway linking plasma membrane receptors to actin filament nucleation, e.g. in chemotaxis. However, lamellipodia are also formed in many cells incessantly during motion with a constant state of the signaling pathways upstream from nucleation promoting factors (NPFs), or spontaneously in resting cells. These observations strongly suggest protrusion formation can also be a consequence of the dynamics downstream from NPFs, with signaling setting the dynamic regime but not initiating the formation of individual protrusions. A quantitative mechanism for this kind of lamellipodium dynamics has not been suggested yet. Here, we present a model exhibiting excitable actin network dynamics. Individual lamellipodia form due to random supercritical filament nucleation events amplified by autocatalytic branching. They last for about 30 seconds to many minutes and are terminated by filament bundling, severing and capping. We show the relevance of the model mechanism for experimentally observed protrusion dynamics by reproducing in very good approximation the repetitive protrusion formation measured by Burnette et al. with respect to the velocities of leading edge protrusion and retrograde flow, oscillation amplitudes, periods and shape, as well as the phase relation between protrusion and retrograde flow. Our modeling results agree with the mechanism of actin bundle formation during lamellipodium retraction suggested by Burnette et al. and Koestler et al.

## Introduction

The crawling of many different cell types is essential for life. In the developing embryo, undifferentiated cells move towards a site, where they form a tissue or organ. Immune cells like neutrophils squeeze through the walls of blood vessels and crawl towards the site of an infection. Skin cells start crawling when they have to close a wound [1]. During metastasis, cancer cells dissociate from the primary tumor, crawl towards blood vessels and spread all over the body [2], [3]. In vitro, cells are typically plated on a two dimensional substrate in order to investigate their motion. It is observed that cells form a flat membrane protrusion in the direction of motion, the lamellipodium, which is usually only about 200 nm thick but several µm long [4].

The motion of these cells is driven by the dynamics of the cytoskeletal actin filaments. A dense network of branched actin filaments pushes the leading edge membrane forward [5]. The filaments can can generate force since they treadmill, which means that the barbed or plus ends polymerize at the leading edge of the lamellipodium, and the pointed or minus ends depolymerize at the rear [6]. When growth factors bind to membrane receptors, they stimulate signaling cascades that lead to the activation of nucleation promoting factors (NPFs) (like WASp or WAVE), which activate the actin related protein complex Arp2/3. Arp2/3 initiates the growth of a new filament branch from an existing filament. The plus end growth can be terminated by the binding of capping proteins. Actin depolymerization factor (ADF) or cofilin severs actin filaments upon binding and enhances depolymerization at the rear [6]. The actin network has to be stabilized by attachment of cross-linking proteins for efficient transmission of force to the leading edge membrane. Further away from the leading edge, actin filaments form a cross-linked gel and are often arranged in bundles or arcs of long filaments in a part of the cell that is referred to as the lamella.

Different cell types can have very distinct shapes and exhibit different “modes” of motion. Fish keratocytes with a stable crescent shape and a broad lamellipodium migrate fast and uniformly [7]. In contrast, the social amoeba *Dictyostelium discoideum* protrudes and retracts pseudopodia in all directions, and moves in a more random fashion towards a chemoattractant [8]. “Pseudopodia” is a more general term for actin rich membrane protrusions of different morphologies, and in the case of Dictyostelium, they are thicker and less broad than lamellipodia. Keratocytes with less regular and smooth-edged morphologies also show less persistent motion [9]. Cycles of protrusion and retraction are thought to help the cell exploring the chemical and mechanical properties of its environment [10]. If a lamellipodium protrudes into favorable surroundings, it can be stabilized and leads to motion in this direction [11].

Distinct cycles of protrusion and retraction have been observed at the edge of stable lamellipodia of spreading and motile cells (reviewed in [10], [12], [13]). A variety of spreading cells exhibit lateral waves traveling around their circumference [14] or oscillatory motion of the leading edge [15], [16]. Machacek and Danuser [17] find other characteristic “morphodynamic patterns” in motile cells, like synchronized retraction and protrusion (“I-state”), or random bulges splitting and traveling along the leading edge of a lamellipodium laterally in different directions (“V-state”). Those patterns are found in a variety of cell types, and can change upon Rac1 activation in epithelial cells. When Dictyostelium is exposed to short pulses of the chemoattractant cyclic AMP, damped or maintained oscillations of the cortical F-actin density with a resonance period of 20 are observed [18].

Patterns are not restricted to the edge of existing lamellipodia, but the whole lamellipodium can be dynamic as well. Upon PAK3 depletion, a lamellipodium has been observed to travel around a circular *Drosophila* cell [19]. Burnette et al. [20] monitor the structure of the actin network in epithelial cells during subsequent cycles of protrusion and retraction and show that the lamellipodium evolves into the lamella during retraction. Similar observations have been made with melanoma cells [21]. The duration of those cycles that involve the whole generation and collapse of the lamellipodium is about 10 minutes [20], in contrast to one to two minutes of the I-state of the lamellipodium edge [17]. Still different phenomena observed in Dictyostelium [22] and neutrophils [23] are waves of high density of filamentous actin (F-actin) traveling along the ventral membrane attached to the substrate that lead to the formation of a protrusion when impinging on the cell perimeter [24].

The origin of this multifaceted dynamics has not been explained yet. The discussion has both biological and mathematical aspects. In mathematical terms, the V-state of Machacek et al. [17], [25] and the morphodynamics in XTC cells [26] have been identified with excitable dynamics. The term excitable refers to the response of a system to perturbations. An excitable system responds to supercritical perturbations with a strong amplification into an excited state, but not to subcritical ones. The most popular example is the action potential spike of an excitable neuron, where the rest state is the polarized membrane. The perturbation arises typically from postsynaptic excitatory currents elicited by other neurons, and the amplification is the depolarization to a full action potential spike. Here, we will show that cycles of protrusion and retraction of the whole lamellipodium can also be described in terms of excitable dynamics. In the model, the rest state does not have a lamellipodium. The perturbation is the formation of a few free plus ends able to polymerize and the amplification is the generation of a lamellipodium. After some time in the excited state, followed by a refractory period during which the excitation threshold is very high, the system returns to the rest state completing the excitation loop.

Repeated generation of lamellipodia requires repeated perturbations. We show that the noise inherent to all cellular processes may suffice. If the excitation threshold of the excitable system is larger than the typical noise amplitude, supercritical perturbations are rare, and the time between excitations is irregular [27]. In the opposite case, where the typical noise amplitude is large compared to the excitation threshold, an excitation arises as soon as the refractory period of the previous one has passed, and the sequence of events is almost as regular as with (limit cycle) oscillations. Excitable systems often exhibit a transition towards maintained oscillations upon a parameter change causing the disappearance of the perturbation threshold. The system then re-enters the excitation loop as soon as the refractory period is over. The transition between the V- and I-state of the morphodynamic phenotypes can be described by the transition from an excitable to an oscillatory regime [25].

The interesting question from a biological point of view is, how excitability in lamellipodium dynamics is realized, since in principle it can be created by many different parts of the system [28]. Several hypotheses have been formulated mainly through mathematical models. The excitability may either be in the actin filament dynamics determined by polymerization, capping, severing, cross-linking, membrane binding, and depolymerization, with constant concentrations of the proteins controlling them (NPFs, cofilin, capping protein, Arp2/3 etc.) as in refs. [25], [29]–[32] for the morphodynamics of existing protrusions and actin density waves at the ventral membrane of Dictyostelium. Or it could be in the signaling pathways or feedbacks converging onto these proteins like in refs. [26], [33]–[35], see [10], [13], [36], [37] for reviews. Many of these latter models have been developed to describe chemotaxis. In these cases, the perturbation causing a local excitation of signaling pathways is the extracellular gradient of the chemoattractant. In ref. [38], an excitable system, coupled to a ‘local excitation global inhibition’ (LEGI) module, was assumed to describe chemotaxis without the assignment of specific molecular mechanisms.

In contrast to the assumption of signaling initiating protrusions like in chemotaxis, protrusions form and retract also with a constant state of the pathways upstream of NPFs and therefore constant fraction of active NPFs. B16 melanoma cells exhibit transitions forth and back between protrusion and retraction without any indication of oscillating signaling molecule concentrations [21]. The data support a mechanism formed by filament polymerization, reorientation, capping, severing, and incorporation into the lamella. Essentially the same mechanism has been described for PtK1 cells [20]. Here, we present a mathematical model that exhibits excitability in the actin nucleation dynamics, accounting for transient lamellipodium formation. We fit the experimentally measured data from Burnette et al. [20], reconstitute the formation of actin bundles in the lamellipodium, and confirm the oscillation mechanism suggested by Burnette et al., which has also been previously described by Koestler et al. [21].

The results presented here are the continuation of the analysis of a modeling framework that has been used to explain a variety of phenomena related to actin dynamics [25], [29], [39], [40]. The derivation of the extension of the model in the form used here has been described in detail in ref. [41], where we also discuss conditions for the existence of stable lamellipodia and oscillations at the leading edge of stable lamellipodia [41]. Here, we show that the same modeling framework can account for the generation of the whole lamellipodium and its retraction, as e.g. reported in [19]–[21]. As the different length and time scales suggest, the repetitive lamellipodium formation is fundamentally different from the oscillatory edge dynamics of stable, existing lamellipodia.

## Results

### Main Features of the Model

**Figure 1:**
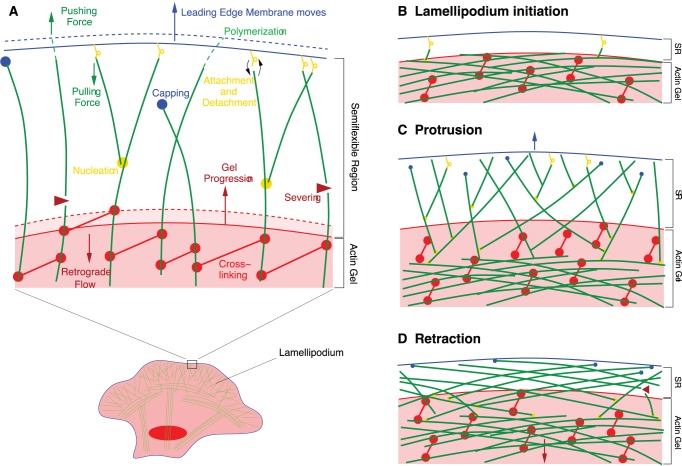
Semiflexible region and actin gel in the model. (A) Schematic representation of processes defining the model. The actin filaments (*green*) in the semiflexible region (SR) can bend, if they are sufficiently long. They exert forces on the leading edge membrane (*blue line*) and push it forward. They elongate by polymerization and shorten by attachment of cross-linkers (*red dumbbells*). Cross-linking also advances the gel boundary (*red line*) defined by a critical concentration of bound cross-linkers. Retrograde flow in the actin gel counteracts forward motion of the gel boundary. Filaments can also attach to the leading edge membrane and exert a pulling force. New filaments are nucleated from attached filaments. Filaments can get capped or severed and then vanish into the gel due to cross-linking or bundling of bent filaments. (B–D) Changes in SR structure during cycles of protrusion and retraction. (B) The formation of a transient lamellipodium is initiated by nucleation of single short filaments from actin bundles in the gel. (C) An actin network grows due to branching, the filament density in the SR increases and the leading edge protrudes. (D) As the filaments in the SR get longer, capping and severing rates increase, the filament density goes down and the lamellipodium retracts. While the SR depth stays narrow, filaments get so long that they have to bend and are likely to form arcs or bundles parallel to the leading edge.

We have developed a mathematical model for lamellipodium protrusion (see Model section for details, ref. [41]). The model lamellipodium consists of an actin gel in the bulk and a highly dynamic range at the leading edge, called semiflexible region (SR) (see Fig. 1 A). The boundary between gel and SR is defined by a critical density of cross-linking proteins bound to the actin filaments. The SR is dynamically maintained since filaments polymerize at the leading edge. Afterwards, cross-linkers bind to the filaments. The filaments can bend in response to forces as long as only a few cross-linkers have bound. They exert a force on the leading edge, which depends on the length of the filaments in the SR and their degree of bending. The longer the free length of filaments, the weaker the force they can exert. The tips of the filaments can also attach to the leading edge membrane. This binding of filaments to the surface against which they push has been shown for the reconstituted systems with beads [42], and oil drops [43], and *in vivo* it is strongly suggested by the observation of leading edge dynamics in mouse embryonic fibroblasts [16]. It is additionally suggested by the presence of a large variety of actin-membrane linking molecules at the leading edge [12], [44]. Attached filaments may not only exert a pushing force when compressed, but also pull the membrane when stretched out.

The density of filaments in the SR increases by nucleation of new filaments from the Arp2/3 complex. When filament plus ends get capped, they stop polymerizing and will quickly vanish into the gel. The same holds when filaments are severed by cofilin. Hence, the filament density decreases by capping and severing. The free filament length in the SR shortens and the gel boundary advances by cross-linking. Forward motion of the gel boundary due to cross-linking is slowed down by retrograde flow, which arises from the force exerted on the gel boundary by the filaments in the SR or contraction in the actin gel, e.g. due to myosin motor activity. The retrograde flow depends on parameters like the friction coefficient of the gel with structures adhered to the substrate, the viscosity of the actin gel, and the active contractile stress in the gel.

With several simplifications, the model describes a one dimensional cross-section through the lamellipodium. While bending of filaments seems to interdict this reduction of spatial dimensions, the schemes in Fig. 1 illustrate that the model assumptions are valid since translational invariance along the gel boundary and leading edge is still given. Averaging over the height of the lamellipodium (thin film approximation) leads to a system of five ordinary differential equations (Eqs. 1–5, typical parameter values in Table 1). They determine the density of attached and detached filaments in the SR, 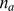 and 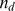, their mean length in the SR, 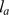 and 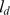, the position of the leading edge and the gel boundary and the distance 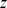 between them, the retrograde flow velocity, and the density 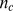 of capped filaments exerting a force. The rest state before protrusion formation, i.e. the absence of a protrusion, corresponds to a state with all filament densities in the SR equal to zero. The state without a lamellipodium exists for all parameter values but is not always the only stable steady state.

**Table 1:** List of model parameters and their values.

Symbol	Meaning	Value	Units
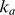	attachment rate of filaments to membrane	10.0	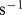
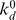	detachment constant	25.0	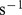
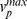	saturation value of polymerization velocity	36.0	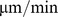
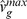	saturation value of gel cross-linking rate	0.03	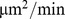
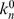	nucleation rate	2.0	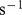
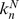	limiting factor of nucleation rate	0.0016	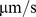
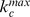	capping rate	1.2	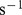
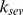	binding rate of cofilin	2.0	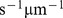
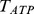	average life time of ATP-actin within filament	8.66	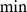
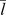	saturation length of cross-linking rate	10	1
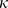	drag coefficient of plasma membrane	0.113	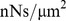
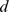	actin monomer radius	2.7	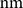
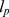	persistence length of actin	15	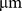
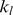	spring constant of linker protein	1	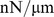
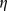	viscosity of actin gel	0.833	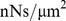
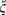	friction coefficient of actin gel to adhesion sites	0.175	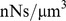
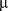	active contractile stress in actin gel	0	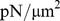
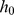	height of lamellipodium at leading edge	0.25	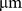
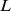	length of gel part of lamellipodium	10	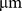
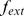	external force on leading edge	0	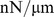

**Figure 2:**
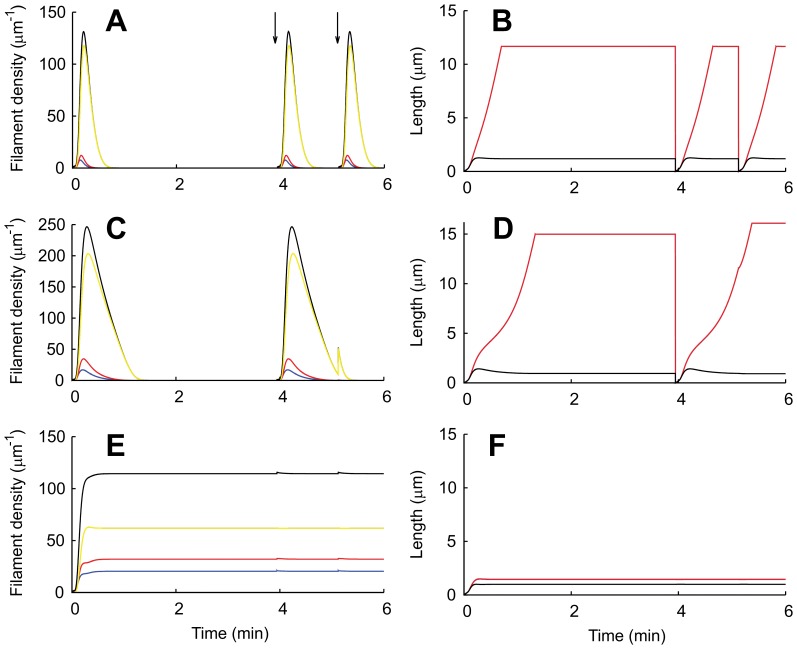
Solutions of the model describing transient and stable lamellipodia. (A–D) Simulations in the excitable regime with stationary filament density n = 0. At random time points (arrows, 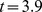 min, 5.1 min), the density of attached filaments is incremented by one, which corresponds to random nucleation of a filament from the cortex or from filaments oriented parallel to the leading edge. Random nucleation of one filament corresponds to a supercritical perturbation of an excitable system. The transient increase in filament density describes lamellipodium formation and collapse. (A, C, E) Density of attached (blue), detached (red) and capped (yellow) filaments and total filament density (black). (B, D, F) Filament length and SR depth (black). Attached (blue) and detached (red) filaments are almost equally long so that both lines overlap. (B, D) The SR depth remains constant as the filaments grow very long. Consequently, they have to bend and form arcs. (A, B) With the parameters from Table 1, (C, D) with 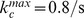, all other parameters unchanged. Decreasing the capping rate has a similar effect as increasing the nucleation rate (see Fig. 3). Filament densities and duration of transients increase. The second increment does not lead to a transient lamellipodium formation in (C, D) because the filament density has not dropped below 1/µm yet and filament length is not decreased. (E, F) 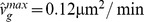, all other parameters as in A, B. The lamellipodium is stabilized since faster cross-linking prevents the filaments from getting too long and floppy.

**Figure 3:**
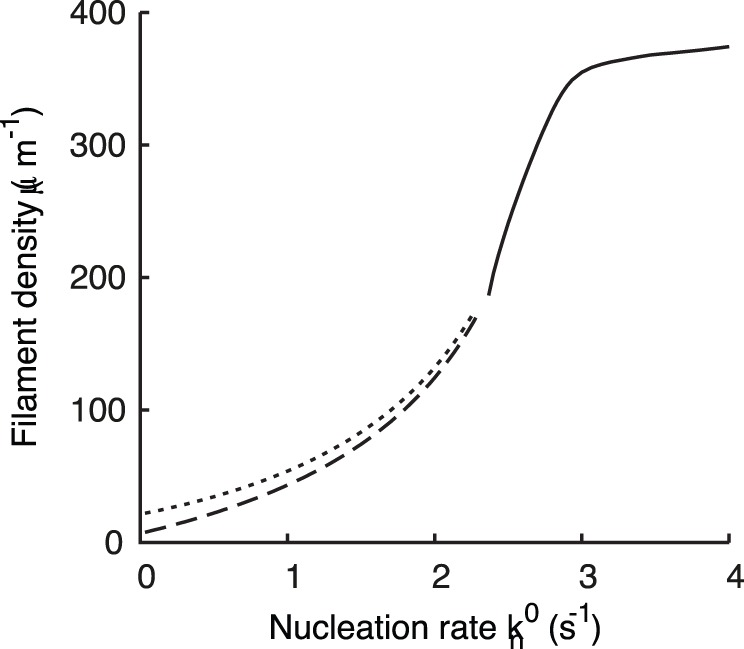
Maximum filament density of the transient lamellipodium in the excitable regime as a function of the nucleation rate. (Dashed line) With 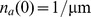 and 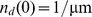 as initial conditions. (Dotted line) With 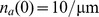 and 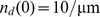 as initial conditions. 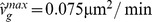, 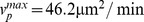, all other parameters as in Table 1. At 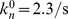, a transition to a stable lamellipodium takes place. (Solid line) Value of the filament density of the stable fixed point existing above this. The leading edge protrusion velocity is proportional to the filament density because the gel cross-linking velocity is proportional to the filament density.

If the nucleation rate is low, nucleation of new filaments cannot compensate for capping and severing. No stable lamellipodium can exist and the absence of a protrusion is the only stable stationary state. However, we can still observe a transient increase in filament density, corresponding to a transient protrusion, when calculating the solution of our dynamical system in that parameter regime (see Fig. 2 A–D). Increasing the nucleation rate has two stabilizing effects. Faster nucleation makes it harder for capping and severing to decrease the filament density. Additionally, the cross-linking rate increases with increasing filament density (Eq. 10). Faster cross-linking slows down the growth of free filament length in the SR, which keeps filaments stiff and the severing rate low (see Eqs. 1, 2). Thus, the filament density stays at a stationary value and the protrusion persists (see Fig. 2 E, F). The transition from a transient to a stable protrusion with increasing nucleation rate is marked in Fig. 3 by the transition from a dashed/dotted to a solid line. The transient lamellipodia in Figs. 2 A–D can also be stabilized by only increasing the cross-linking rate as in Figs. 2 E, F. A higher cross-linking rate stabilizes the lamellipodium without increasing the filament density by the mechanism explained above. (See ref. [41] for additional phase diagrams and a more comprehensive description of existence conditions for stable lamellipodia.).

The role of the cross-linking rate for stabilizing protrusions illustrates the necessity for the mechanical support to keep up with polymerization in order to maintain steady protrusion. Without sufficiently fast gel formation, filaments in the SR grow long, become floppy, cannot push anymore, motion ceases and polymerization speeds up due to the decrease of forces. This is all illustrated by the transient lamellipodia described in the next section.

### Excitability Mechanism for Transient Lamellipodia Formation

Solutions corresponding to the transient formation of a lamellipodium are shown in Fig. 2 A–D. We have incremented the density of attached filaments by one at random time points, to model the random nucleation of single filaments from the actin cortex or from actin bundles oriented parallel to the leading edge. Vinzenz et al. [45] provide experimental evidence that lamellipodia can indeed initially form by such a mechanism. The observations are made with holes punched with a microneedle into the lamellipodia of B16 melanoma cells, 3T3 fibroblasts, or keratocytes. Monitoring the hole edge in the electron microscope while it heals shows that short filaments branch from long filaments that are oriented parallel to the edge of the hole. The newly nucleated filaments initiate the growth of a dendritic actin network filling the hole.

In our simulations, newly nucleated filaments are short, and short filaments exert large forces (see Eq. 15). These forces drive the leading edge membrane forward and the width of the semiflexible region increases (Fig. 2 B, D). According to its force-dependence (Eq. 8), the capping rate is low for short filaments. It is also unlikely that short filaments get severed, since cofilin preferentially binds to ADP-actin [46]. When monomers bind to the plus end, they have ATP bound. Before cofilin can bind, the ATPase activity of actin needs to hydrolize ATP and the phosphate has to dissociate from actin. That causes an increase of the binding rate with polymer length (see the expression for the severing rate in Eqs. 1, 2). Consequently, the number of filaments grows due to nucleation at early times (Fig. 2 A, C).

As the filament density increases, filaments grow longer (Fig. 2 B, D) and exert weaker forces. The capping and the severing rates now both increase because of their length dependencies. Filaments also disappear from the SR by incorporation into the gel. Capping, severing and cross-linking finally lead to the falling phase of the filament density. Filaments keep polymerizing while the density decays exponentially in time. The exponential decay entails that the filament density never becomes exactly zero. We introduce a cut off at one filament/µm at which we set the polymerization velocity 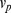 to zero to avoid fractions of filaments with unreasonable length. This cut off leads to the plateaus in filament length in Fig. 2 B, D.

As filaments get longer during the phase of decreasing filament density, they polymerize even faster since weak forces entail a large polymerization rate (Eq. 9). While the filaments grow long, the SR depth 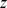 remains unchanged and narrow. Since the filaments get much longer than 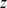 (Fig. 2 B), they only fit into the SR when they bend and most of their length orients parallel to the leading edge (see Fig. 1 D). This filament reorientation exactly recapitulates the mechanism of actin arc formation during lamellipodium retraction described in refs. [21] and [20].

A random increment of the filament density at the end of the second protrusion in Fig. 2 C illustrates the function of filament length in protrusion generation. We can assume that at this time the new filaments are not nucleated at the gel boundary but from the mother filament at some distance from the graft point. They do not have their own mechanical support and consequently do not contribute to the strength of the SR. When their minus ends reach the gel boundary, the new filaments gain mechanical support. However, they are not as short as the filaments at the beginning of protrusion generation and therefore cannot exert strong forces. Hence, nucleation of single filaments only leads to a slight increase in filament density in the existing protrusion and fails to initiate a new protrusion.

In Fig. 3, the amplitude of the transient lamellipodium starting from the resting state is shown as a function of the nucleation rate. The maximum filament density increases with increasing nucleation rate until the lamellipodium becomes stable. As the filament density in the transient lamellipodium increases, it takes also longer until it vanishes again. Decreasing the capping rate has a similar effect as increasing the nucleation rate (see Fig. 2 C, D).

### Fit to Experimental Data from Epithelial Cells

We compared our model to the cycles of protrusion and retraction measured by Burnette et al. [20]. The authors distinguish lamella and lamellipodium and discuss in detail how the actin network of the lamellipodium evolves spatio-temporally into the lamella during the retraction phase of edge motion. The differentiation between lamellipodium and lamellum is not only based on different actin structures (branched networks and bundles or arcs) but also associated with adhesion maturation [32] and depolymerization [47]. To avoid confusion we would like to point out that the gel region and the SR defined in our model are not identical with lamella and lamellipodium, resp. The SR is the network region juxtaposed to the leading edge membrane with a (dynamic) width in the range of 1 µm. It is the front region of the lamellipodium. The gel comprises the remaining more retrograde part of the lamellipodium and the lamella.

**Figure 4:**
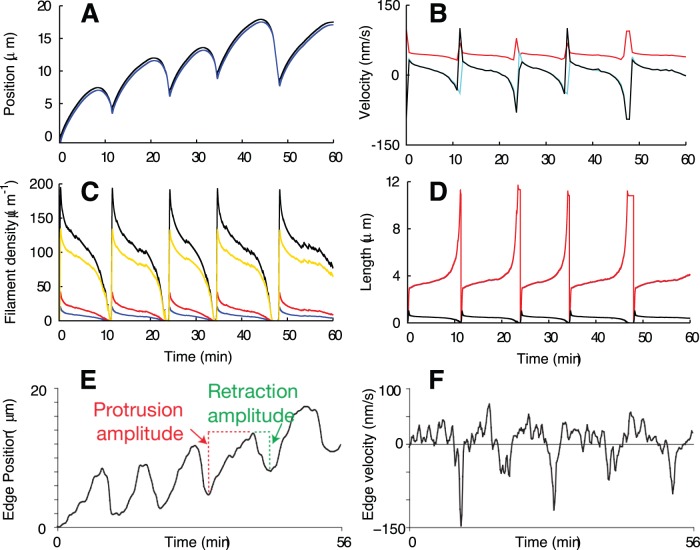
Simulation of the measurements with epithelial cells from Burnette et [20]. The same simulation as in Fig. 2, fitted to the experimental data from Burnette et al. [20] (E, F). Random nucleation occurs more frequently, so that a new lamellipodium forms right after the collapse of the previous one. (A) Position of the leading edge (black) and the gel boundary (blue). The SR depth, which is the distance between leading edge and gel position, is shown as a black line in (D). (B) Velocities of the leading edge (black) and the gel boundary (light blue) and retrograde flow velocity (red). (C) Density of attached (blue), detached (red) and capped (yellow) filaments and total filament density (black). (D) Filament length and SR depth (black). Attached (blue) and detached (red) filaments are almost equally long so that both lines overlap. Parameters are 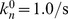, 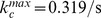, 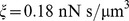, 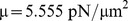. Membrane tension is characterized by an external force 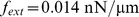. All other values like in Table 1. (E) Experimentally measured leading edge position (Fig. 5b from [20]). (F) Measured leading edge velocity (Fig. 5a from [20]. E, F published with permission from Nature Cell Biology.

The model was fit to the time course of membrane position reported in Fig. 5b of ref. [20] (Fig. 4 E). The result is shown in Fig. 4 A–D. Nucleation of single filaments corresponds to superthreshold noise of the excitable system. Random nucleation occurs more frequently than in Fig. 2 so that a new lamellipodium forms right after the collapse of the previous one. The leading edge time course (Fig. 4 A) compares very well with the measured data (Fig. 4 E), with the protrusion amplitude of 5–10 µm as well as the duration of one cycle of around 10 min. Consequently, also the time courses of leading edge velocities are in agreement between experiment (Fig. 4 F) and simulation (Fig. 4 B). Also in accord with the experiment (Fig. 1f in [20]), the retrograde flow goes up, before retraction of the lamellipodium is finished (Fig. 4 B). The acceleration of retrograde flow can therefore not be due to an increase in total force on the leading edge membrane.

**Figure 5:**
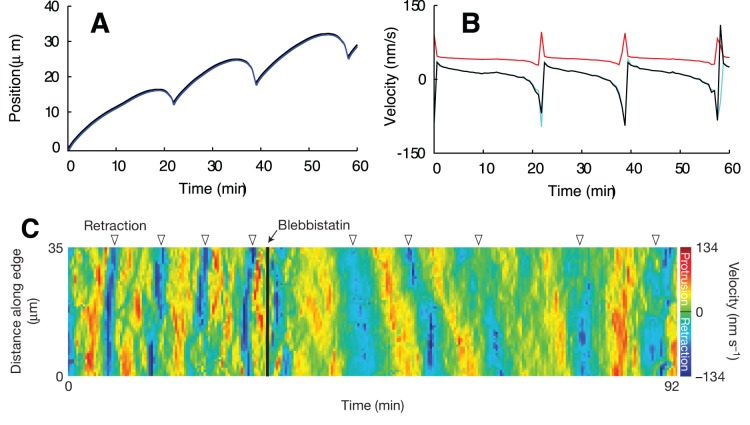
Simulation of myosin inhibition. The same simulation as in Fig. 4, but with a myosin contractility 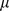 of 0.225 pN/µm^2^ instead of 5.555 pN/µm^2^. (A) Position of the leading edge (black) and the gel boundary (blue). (B) Velocities of the leading edge (black) and the gel boundary (light blue) and retrograde flow velocity (red). Comparison with Fig. 4 shows that the measured increase in period (C) is reproduced by our simulation. Filament densities, filament lengths and SR depth show basically the same behavior as without myosin inhibition, see Fig. 4 C, D. (C) The measured velocity map from Burnette et al. (Fig. 4 from [20]) shows that the period of protrusion cycles increases when myosin is inhibited by application of Blebbistatin. Published with permission from Nature Cell Biology.

Note that this phase relation between retrograde flow and membrane velocity was not subject to the fit and is therefore an independent confirmation of the agreement of the model with the experiments. This phase relation might be specific to the excitable protrusion generation dynamics, since it was not observed in systems exhibiting oscillatory dynamics of existing protrusions [16], [26], [41]. The increase of retrograde flow during edge retraction arises from the dependency of the factor 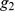 in Eq. 23 on the cross-linking rate 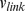. This dependency is the result of a semi-analytic solution [48] of the gel equations derived by Kruse et al. [49] in a quasi-stationary approximation, which we slightly modified here to avoid singularities. The original derivation suggested, that the quasi-stationary approximation might not be valid for very small 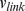, i.e., in the limit of very few filaments. Here, we find that exactly the small-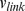 behavior reproduces the measured phase relation. Hence, the semi-analytic solution presented in ref. [48] might be broader applicable than originally assumed.

The model suggests a mechanism of actin arc formation during retraction, which is also essentially the same as suggested by experiments. Each cycle of protrusion and retraction forms one arc and mainly during retraction. As the lamellipodium collapses, the filaments grow very long while the width of the SR shrinks (Fig. 4 D). They have to orient parallel to the leading edge, thus assuming a position favorable for bundling by myosin (see Fig. 1 B–D and drawings in refs [20], [21]). Arc formation occurs in our model in the semiflexible region, i.e. within about 1 22 m of the leading edge membrane. The length scales are in good agreement with the data presented in Burnette et al. [20], Fig. 3b and Movie S3, and the ideas presented in Koestler et al. [21], Fig. 5. Arcs are embedded into the gel as the gel boundary moves forward. Consequently, they will finally be embedded into the lamella.

In [20], it was also shown that the cycles of protrusion and retraction are independent of myosin activity and arc formation. Application of Blebbistatin, a myosin inhibitor, led to the loss of arcs, disruption of the apparent boundary between lamellipodium and lamella, and larger amplitudes and longer durations of the transient protrusions (see Fig. 4 in [20], Fig. 5 C). Myosin inhibition can be simulated in our model by simply reducing the active contractile stress in the actin gel 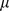 (Fig. 5 A, B). The amplitude increases from 5–10 µm to 12–14 µm and the period from about 12 min to about 18 min. In fact, since in the simulations of transient protrusions shown in Fig. 2 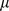 was set to zero, contractile stress in the gel is not necessary in the model for observing subsequent lamellipodium formation and collapse. However, for 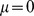, no backward motion of the leading edge is observed.

## Discussion

We have presented a mechanism that is based on excitability in concert with random filament nucleation, and offers an explanation for irregularly and regularly repeated protrusion formation. We were able to predict that periodic lamellipodium formation is determined by the autocatalytic nature of branching, and the length dependence of bundling, capping and severing. The initiating nucleation (Fig. 1 B) leads to lamellipodium formation by a transient increase in filament density due to branching (Fig. 1 C), upon which the filament density decreases due to bundling, capping and severing, and the lamellipodium collapses. Our model also predicts the formation of actin arcs. As the number of filaments decreases, the remaining filaments grow longer (Fig. 1 D). They have to bend since the depth of the SR remains small. This mechanism describes very well the protrusion and retraction of lamellipodia in PtK1 epithelial cells observed by Burnette et al. [20]. They show that actin arcs form in the lamellipodium at the peak of the protrusion phase and are then retracted and incorporated into the lamella. Being oriented parallel to the leading edge, they serve as the base for the protrusion of a new lamellipodium. The same mechanism has been previously described for B16 melanoma cells [21]. Our simulations support the finding that actin arcs of the lamella form at the leading edge and provide a “stiff substrate for actin filaments in the lamellipodium to push back against to extend the plasma membrane” [20].

An important conclusion from the model is that there is a mechanism for which no cell signaling is necessary to initiate individual protrusions and retractions. In our model, nucleation, polymerization, capping, and severing rates only change due to varying filament lengths and forces. Neither the concentrations of signaling molecules like active small GTPases nor actin regulators like NPFs or Arp2/3 change during cycles of protrusion and retraction. That such a mechanism exists is suggested by several experiments. Fibroblasts frequently form new protrusions while migrating in a constant chemoattractant gradient [11]. Active Rac1 activates WAVE to stimulate filament nucleation by Arp2/3 and protrusion formation [50], [51]. However, the time course of Rac activation in several experiments seems not to be compatible with Rac being part of an excitable signaling pathway, the excitation of which triggers protrusions. The response of mouse embryonic fibroblasts to a PDGF-BB gradient is a transient rise of activated Rac1, followed by a decrease to base level after 20. However, cells continue to form protrusions for hours [52]. Constitutively active Rac decreases directionality and promotes random motility in fibroblasts [53]. The loss of directionality is due to frequent formation of lateral lamellipodia [11], [53], [54]. Also PI3K signaling upstream of Rac stabilizes existing lamellipodia, but its inhibition does not reduce the frequency of lamellipodium initiation [54]. These scenarios are rather compatible with signaling setting the parameter values for an excitable regime of the actin filament dynamics downstream from NPFs, than with signaling dynamics initiating the formation of each lamellipodium. Our model offers a mechanism for such an excitable protrusion formation. Note, while our mechanism does not require fluctuations in the state of signaling pathways, it would still allow for them initiating protrusion formation. Hence, it is not in conflict with protrusion initiation by signaling as e.g. in chemotaxis.

Different sets of parameters in the model correspond to different cell types or different states of signaling pathways within one cell type. The model predicts that decreasing the nucleation rate leads to a transition from a stable lamellipodium to a transient lamellipodium that exhibits cycles of protrusion and retraction. If the nucleation rate is further decreased, the filament density in the transient lamellipodium decreases along with the protrusion amplitude and the duration of cycles. Finally, lamellipodia will be lost completely. Such a behavior should for example be observed if Arp2/3 is gradually inhibited in an initially stable lamellipodium. This scenario also fits observations of the coexistence of spontaneous transient lamellipodia and stable protrusions in fibroblasts as reported by Welf et al. [54]. Spontaneous protrusions form frequently in these cells [54], which corresponds to the rest state perturbed by noise in terms of the model. Some protrusions develop sufficient local PI3K signaling (once they exist) to be stabilized [54]. PI3K signaling activates Rac and consequently WAVE which increases the nucleation rate [50], [51]. Again in model terms, that provides for the (local) increase in 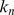 causing the transition into a regime with stable lamellipodia. A transition from a stable lamellipodium to a transient lamellipodium circling the cell has also been observed in *Drosophila* cells upon PAK3 depletion [19]. PAK is thought to activate filamin and inhibit cofilin and myosin [55]. Filamin is a cross-linker and decreasing the cross-linking rate in our model leads to longer, more floppy filaments and a transition from a stable to a transient lamellipodium. The same transition is observed by increasing the severing rate or active contractile stress in the gel, which might both be a consequence of PAK3 depletion. All those possible effects of PAK are in accord with our model predictions.

We expect that the excitability observed in our one-dimensional model gives rise to a wave of high actin density that circles the cell periphery [19], if the model would be extended to two dimensions, since the rise in filament density can “infect” neighboring regions around the cell circumference. A positive feedback, a spreading mechanism and a delayed negative feedback are necessary for wave propagation [28], [56]. Positive and negative feedback are defined in our model by the presented excitability mechanism. As in simulations of F-actin density waves traveling along the ventral membrane of Dictyostelium by Carlsson [30], autocatalytic branching of actin filaments gives the positive feedback. The spreading mechanism could also be the same as in ref. [30]: filaments branch from the mother filament under a certain angle and grow into neighboring regions. In contrast to ref. [30], filament elongation and bending not only provide the negative feedback but additionally account for arc formation in our model.

The characteristic feature of our model is the inclusion of the semiflexible region, a region where the degree of cross-linking is too low for gel-like behavior. This description of the leading edge arises naturally from a view of cross-linking dynamics where free cross-linkers bind to filaments, bound cross-linkers are transported rearward by the retrograde flow of F-actin, and then dissociate and diffuse back towards the front. An important consequence is that filaments lacking the stabilization of cross-linkers are able to bend. This is captured in the model by including the filament free length as a dynamic variable. While the existence of a semiflexible region is a topic of ongoing discussion, this theoretical framework is both suggested from first principles and able to quantitatively describe a wide array of phenomena related to actin dynamics. Analytical solution of a reaction-diffusion description of cross-linker dynamics yields a decreasing gradient in the degree of cross-linking toward the leading edge (see also Eq. 10). Additionally, a weakly cross-linked region emerges from simulations of the same process applied to myosin [31], [57]. Recently, application of this theoretical framework led to suggestions for quantitative explanations of the force-velocity relation of fish keratocytes [39]. By suggesting periodic stress formation that leads to detachment and re-attachment of actin filaments to leading edge membrane, these models are able to explain the experimental observations of velocity oscillations of actin propelled oil drops [29], [40] and beads [58], and morphodynamic phenotypes [25]. The same sequence (stress, detachment, re-attachment) has been used to explain the leading edge dynamics observed in mouse embryonic fibroblasts [16]. All these previous studies considered the dynamics of existing protrusions. In this paper, we were able to extend the explanatory power of the model to protrusion formation dynamics by including nucleation, capping, and severing. The ability of this model to reach quantitative agreement with experimentally observed periodic lamellipodium formation adds further evidence toward the theoretical framework assuming the existence of a semiflexible region at the leading edge.

## The Mathematical Model

The semiflexible region (SR) dynamics is described by the following system of ordinary differential equations [41]:

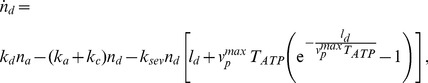
(1)

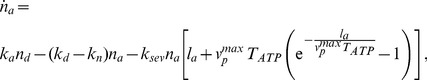
(2)

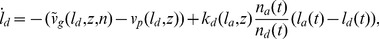
(3)

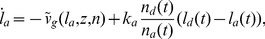
(4)

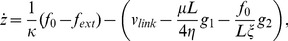
(5)with the density of attached and detached filaments 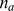 and 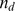, the mean length of attached and detached filaments 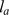 and 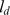, and the SR depth (distance between gel boundary and leading edge membrane) 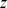. Noise is included into the system by incrementing 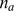 by one at random time points. If the total filament density 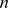 has dropped below 1/µm at that time, the filament lengths 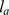, 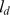 and the SR depth 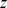 are also set back to their short initial values. The tips of detached filaments can attach to the membrane at a constant rate 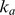. The detachment rate
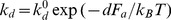
(6)depends on the force exerted by an attached filament 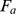 since a pulling force facilitates detachment. The actin monomer radius is denoted by 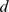, 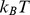 is the thermal energy and 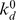 the detachment rate at zero force.

New filaments are nucleated from attached filaments, reflecting the membrane binding mechanism of filaments via NPFs and Arp2/3. Nucleation without negative feedback would lead to an exponential growth of the filament number. Negative feedback may arise from a limited number of Arp2/3 complexes [26] or other limitations. Independent from specific assumptions, we obtain in first order the nucleation rate
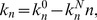
(7)with the constants 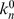 and 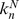. Detached filaments can get capped. The capping rate
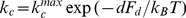
(8)decreases with increasing force 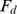 exerted by a detached filament on the membrane. The larger this force, the lower the probability that the tip of the filament fluctuates away from the membrane and a capping protein can attach. This force dependency of the capping rate and its similarity to the force dependency of polymerization have recently been confirmed experimentally [59]. Detached filaments grow by polymerization. Since also the probability of monomer attachment decreases with increasing pushing force, the polymerization velocity
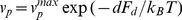
(9)shows the same force-dependence as the capping rate. We set 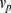 to zero when the filament density 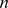 drops below 1/µm. The assumption that attached filaments do not polymerize excludes formins as the polymerization mechanism from the model. However, one could simply add polymerization in Eq. 4 for the 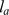 dynamics to include them, too.

The filament density may also decrease due to severing of filaments by cofilin. Cofilin only binds to ADP-actin within a filament [46], hence some time after monomer binding to the tip when the attached ATP has been hydrolyzed. The severing rate is filament length dependent since long filaments have more older parts. 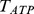 is the average life time of ATP within the filament. The severed filaments will be shorter than 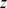, so that they do not exert force and will eventually vanish into the gel. The binding rate of cofilin to ADP-actin is denoted by 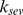.

The gel advances due to cross-linking. The gel boundary is defined by a concentration of cross-linking molecules bound to the actin filaments above which the network has gel-like properties. We have calculated the cross-linking velocity from reaction-diffusion equations of cross-linkers and it depends on the filament length 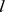 and the filament density 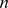 in the following way [48]:
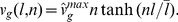
(10)


The characteristic length 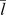 and the maximum cross-linking rate 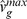 are parameters. In the rate of filament shortening
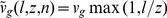
(11)the additional factor 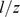 accounts for the fact that a larger portion of filament length is incorporated into the gel during cross-linking when filaments are bent.

The balance of filament and counteracting forces determines the velocity of the leading edge membrane. All viscous forces resisting motion are included in the coefficient 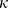. The external force 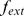 may represent a resisting force due to membrane tension or exerted by an obstacle in the environment. The total filament force reads
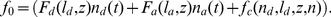
(12)


Detached filaments exert an entropic force on the membrane. Attached filaments can either exert a pushing force when compressed or a pulling force when stretched out. The pushing force is different from the force of detached filaments since the tip of the attached filament is not freely fluctuating. Both forces depend on the filament length and the SR depth, hence on the degree of bending. The entropic force of detached filaments of contour length 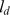 grafted at one end on an obstacle at distance 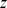 has been calculated in [60] as
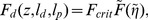
(13)with the scaling variable
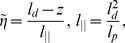
(14)and the critical force for the Euler buckling instability



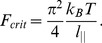
(15)The persistence length of the polymer is denoted by 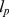. In [60], it is shown that for small compression 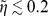 the scaling function of the entropic force can well be approximated as
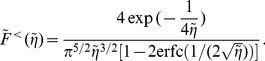
(16)


For 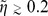 the calculation yields
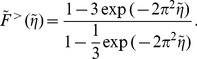
(17)


To calculate the total force of all capped filaments, we have assumed a stationary length distribution of capped filaments [41]. We found that in good approximation
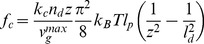
(18)holds. The proteins attaching the filaments to the membrane are assumed to behave like elastic springs. We distinguish three different regimes for the force 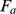 exerted by the serial arrangement of polymer and linker, depending on the relation between the depth of the semiflexible region 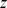, the equilibrium end-to-end distance 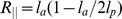, and the contour length 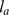 [16]:



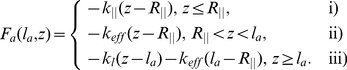
(19)The three cases correspond to: i) a compressed filament pushes against the membrane; ii) filament and linker pull the membrane while being stretched together; iii) a filament is fully stretched but the linker continues to pull the membrane by being stretched further. Here, 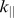, 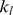 and 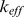 are the linear elastic coefficients of polymer, linker and serial polymer-linker arrangement, respectively. For 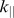 we use the linear response coefficient of a worm-like chain grafted at both ends 
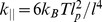
 [62].

The gel boundary advances with the average cross-linking velocity
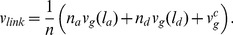
(20)


The contribution of capped filaments
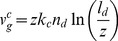
(21)was again calculated assuming a stationary length distribution [41]. This approximation enables us to also calculate the total number of capped filaments



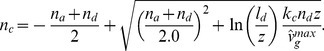
(22)The total number of all filaments in the SR (attached, detached and capped) is then given by 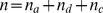. The forward motion of the gel boundary is slowed down by retrograde flow. We have calculated the retrograde flow at the gel boundary as a function of filament forces and the cross-linking rate from the theory of the active polar gel. The first retrograde flow term in Eq. 5 is proportional to the active contractile stress 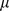 and expresses retrograde flow arising from contraction in the actin network, e.g. due to myosin motor activity. The second retrograde flow term is proportional to the filament force 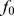 and represents the actin network being pushed backwards due to insufficient adhesion. Adhesion is described as a friction between the gel and the substrate with the coefficient 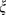 in the gel theory. The additional factors in the retrograde flow terms read
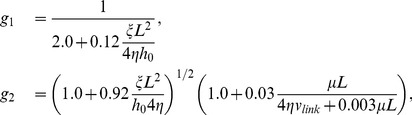
(23)with the viscosity of the actin gel 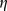, the height of the lamellipodium at the gel boundary 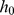 and the length of the gel part of the lamellipodium 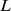.
